# Innovations in Medicine: Exploring ChatGPT’s Impact on Rare Disorder Management

**DOI:** 10.3390/genes15040421

**Published:** 2024-03-28

**Authors:** Stefania Zampatti, Cristina Peconi, Domenica Megalizzi, Giulia Calvino, Giulia Trastulli, Raffaella Cascella, Claudia Strafella, Carlo Caltagirone, Emiliano Giardina

**Affiliations:** 1 Genomic Medicine Laboratory UILDM, IRCCS Santa Lucia Foundation, 00179 Rome, Italy; s.zampatti@hsantalucia.it (S.Z.);; 2 Department of Science, Roma Tre University, 00146 Rome, Italy; 3 Department of System Medicine, Tor Vergata University, 00133 Rome, Italy; 4 Department of Chemical-Toxicological and Pharmacological Evaluation of Drugs, Catholic University Our Lady of Good Counsel, 1000 Tirana, Albania; 5 Department of Clinical and Behavioral Neurology, IRCCS Fondazione Santa Lucia, 00179 Rome, Italy; c.caltagirone@hsantalucia.it; 6 Department of Biomedicine and Prevention, Tor Vergata University, 00133 Rome, Italy

**Keywords:** artificial intelligence, ChatGPT, rare disorders

## Abstract

Artificial intelligence (AI) is rapidly transforming the field of medicine, announcing a new era of innovation and efficiency. Among AI programs designed for general use, ChatGPT holds a prominent position, using an innovative language model developed by OpenAI. Thanks to the use of deep learning techniques, ChatGPT stands out as an exceptionally viable tool, renowned for generating human-like responses to queries. Various medical specialties, including rheumatology, oncology, psychiatry, internal medicine, and ophthalmology, have been explored for ChatGPT integration, with pilot studies and trials revealing each field’s potential benefits and challenges. However, the field of genetics and genetic counseling, as well as that of rare disorders, represents an area suitable for exploration, with its complex datasets and the need for personalized patient care. In this review, we synthesize the wide range of potential applications for ChatGPT in the medical field, highlighting its benefits and limitations. We pay special attention to rare and genetic disorders, aiming to shed light on the future roles of AI-driven chatbots in healthcare. Our goal is to pave the way for a healthcare system that is more knowledgeable, efficient, and centered around patient needs.

## 1. Introduction

Artificial intelligence (AI) is rapidly transforming the field of medicine, announcing a new era of innovation and efficiency. By integrating advanced algorithms and machine learning techniques, AI can enhance diagnostic accuracy, personalize treatment plans, and simplify healthcare operations. This technological evolution is expected to revolutionize patient care, making it more precise, predictive, and personalized than ever before. One of the significant advancements in artificial intelligence is generative AI (GAI), which can be characterized as a system capable of creating various media outputs, such as images, text, and other forms, based on human input [1]. Specifically, GAI models are adept at producing diverse media, including text, images, audio, video, and 3D models, in response to user queries. This cutting-edge technology excels at identifying patterns and analyzing existing datasets to produce outputs that are not only realistic but also consistent with the characteristics of the input. GAI models employ a variety of machine learning techniques, such as deep learning (DL), natural language processing (NLP), and neural networks. Notable GAI systems include ChatGPT, Dall-E, Gemini, and Midjourney. In the realm of healthcare, GAI has the potential to enhance various activities; for example, DALL-E can analyze patient medical images to aid in diagnosis [2]. Similarly, Gemini AI can process images, even with low performance at the moment [3]. Some advanced GAI models are large language models (LLMs) that have been developed to understand and generate human-like languages. The LLMs analyze text using deep learning techniques and generate accurate responses. Among LLMs trained for scientific purposes, there is BioMedLM, a language model developed by Stanford CRFM in partnership with MosaicML. It is specifically trained on biomedical literature (PubMed abstracts and papers) [4] and has demonstrated good performance in biomedicine [5]. Presently, ChatGPT stands out as one of the most prominent LLMs. It uses an innovative language model developed by OpenAI, based in California, USA. Thanks to the use of deep learning techniques, ChatGPT stands out as an exceptionally viable tool, renowned for generating human-like responses to queries. This ability stems from its foundation as one of the Generative Pre-training Transformer (GPT) models, specifically designed to understand, interpret, and generate human language with remarkable proficiency [6,7,8,9,10]. ChatGPT, trained on extensive text datasets, generates outputs that closely mimic the style and content of its training material and maintain relevance and coherence with the input context [11]. Its deep learning basis enables the model to identify patterns in large data volumes, essential for producing accurate predictions and executing specific tasks [12]. At its core, ChatGPT facilitates interactions that resemble human conversation, which is a significant advancement in enabling machines to understand and produce human language in previously unexpected ways. This marks a crucial step in developing more intelligent, responsive, and adaptable AI systems.

ChatGPT became the first large language model achieving broad acceptance and curiosity across the general public [13]. As a chatbot technology, it responds to a diverse array of inquiries, engaging users in dialogues that bear a striking resemblance to natural human conversation. The model gets smarter with each conversation because it learns from its interactions [14,15].

It employs a transformer model, a type of neural network architecture able to analyze the input data, understand the nuances of the language, and map out relationships between words and phrases. Through this process, ChatGPT determines the most appropriate words and phrases to construct a coherent and relevant response in a specific context [16]. The intelligence of ChatGPT lies in its ability to simulate an internet-like breadth of knowledge through its training, enabling it to provide informative and conversationally fluent outputs that are remarkably human-like in their presentation.

Upon its release, it captured the public’s attention, collecting one million registered users within only five days. By the end of two months, this number had risen to over 100 million, underscoring the chatbot’s widespread appeal [17]. While it does not pull information from the internet in real time, the illusion of comprehensive knowledge is crafted through its prior training on an extensive dataset. Its ability to engage in natural, human-like dialogue and provide informative answers with remarkable contextual awareness has fueled its adoption by curious users worldwide.

Furthermore, from September 2023, ChatGPT can browse the internet [18,19]. It generates responses based on a mixture of licensed data, data created by human trainers, and publicly available data, which is then used to pre-train the model. To date, its responses are generated based on a fixed dataset available up to the last training cut-off in April 2023. One of the significant limitations of articles on ChatGPT published before September 2023 is the restricted access of the chatbot only to information before 2021. The update of the ChatGPT in September 2023 allows it to access a more completed and updated dataset (dated April 2023). It is important to note that while ChatGPT can provide varied responses to repeated queries over time, this variation is not due to live internet access or real-time data updates; rather, it is a result of its design to produce non-deterministic outputs. Nonetheless, repetitive questioning at different times might result in different answers with some critical differences in completeness and correctness, as reported below.

**Figure 1:**
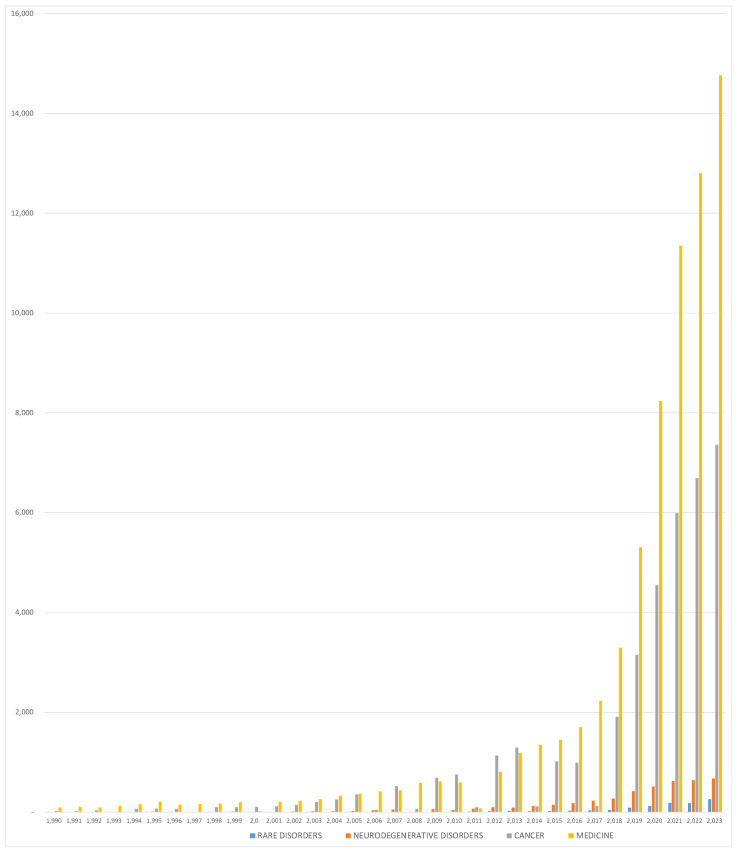
Histogram showing the number of PubMed results per year (“Artificial Intelligence” and, respectively, “rare disorders”, “neurodegenerative disorders”, “cancer”, and “medicine”).

Integrating artificial intelligence (AI) into the healthcare sector signals a new era, characterized by enhanced precision, accuracy, and efficiency. While AI has been widely adopted in domains such as customer service and data management, its use in healthcare and medical research needs careful consideration. The use of AI in healthcare is not only beneficial but crucial, given its capacity to transform medical practices through time efficiency. However, it is equally essential to methodically assess its limitations to prevent unacceptable mistakes and errors in medicine [20]. Despite its considerable potential for applications across various medical fields, AI remains rarely applied to rare diseases. Specifically, an examination of the volume of scientific publications since the 1990s revealed a clear growth in several medical fields, with a slight increase in rare disease (Figure 1).

One of the primary benefits of ChatGPT in the medical domain is its contribution to research and education. With its advanced writing abilities and contextually adaptive language, ChatGPT has the potential to be a powerful tool for synthesizing research, drafting papers, and composing coherent and context-aware literature reviews, though concerns exist regarding its accuracy, potential for misuse, and the ethical implications of its application. Also, ChatGPT can play an essential role in education, though it has some limits. It can help training medical professionals by offering interactive learning and simulating clinical situations for teaching. One of the most promising applications of ChatGPT is its ability to enhance clinical practice. It can provide initial diagnostic recommendations, assist in developing differential diagnoses, and suggest treatment options.

Various medical specialties, including rheumatology, oncology, psychiatry, internal medicine, and ophthalmology, have been explored for ChatGPT integration, with pilot studies and trials revealing each field’s potential benefits and challenges. However, the field of genetics and genetic counseling, as well as that of rare disorders, represents an area suitable for exploration, with its complex datasets and the need for personalized patient care.

In this review, we synthesize the wide range of potential applications for ChatGPT in the medical field, highlighting its benefits and limitations. We pay special attention to rare and genetic disorders, aiming to shed light on the future applications of AI-driven chatbots in healthcare. Our goal is to pave the way for a healthcare setting that is more knowledgeable, efficient, and centered around patient needs.

## 2. Medical Research and Literature Production

The potential applications of AI are extensive, reflecting its adaptability and the depth of its training. In academic contexts, the ChatGPT chatbot was utilized to assist in composing theses, structuring research projects, and drafting scientific articles [21]. This highlights ChatGPT’s potential to facilitate the scholarly writing process, marking it as a significant tool for students, researchers, and academics. ChatGPT has passed the United States Medical Licensing Exam (USMLE), demonstrating its ability to learn and utilize complex medical knowledge to meet high professional standards [22,23].

Despite an expected shortfall in areas requiring high creativity, ChatGPT can be helpful in structuring original research outlines on specific topics. It can deliver a comprehensive research outline that meticulously resembles the structure and detail expected in standard research projects [24]. This demonstrates its understanding of academic norms and its ability to adjust language and output to make them suitable for the context provided. Despite ChatGPT’s ability to draft articles based on selected scientific literature, its feasibility for topics on rare disorders is yet to be confirmed. This is primarily because language models like ChatGPT exhibit frequency bias: they perform better with concepts that are extensively covered in their training data and less so with lesser-known topics [25]. Consequently, the reliability of ChatGPT’s responses is higher for diseases that are more prevalent in the dataset used for pre-training the model (last updated in April 2023) compared to those with less available information. For instance, it has been observed that ChatGPT’s information on common conditions like osteoarthritis and ankylosing spondylitis is more accurate than that on relatively rare diseases, such as psoriatic arthritis [26].

While ChatGPT emerged as a significant supporting tool in drafting medical research articles, its ideal fit for this task warrants a critical examination. The AI’s contribution to manuscript preparation can be helpful, yet the decision by some researchers to list ChatGPT as a co-author on publications suggests caution. This practice raises ethical and practical questions about the nature of authorship and intellectual contribution. Co-authorship traditionally conveys a degree of intellectual investment and responsibility for the content, aspects that AI, by its current design, cannot fulfill. Furthermore, the implications for accountability, especially in fields as sensitive as medical research, are profound. To date (last access on 12 February 2024), PubMed acknowledges four articles that formally list ChatGPT in the authorship [27,28,29,30], while Scopus records three [31,32,33]. These figures are undoubtedly an underestimate. Many papers have acknowledged ChatGPT in the author list during their initial presentation, a fact that is inconsistent with reports from PubMed [33].

Support from chatbots was tested as a replacement for human authors. In June 2023, some scientists even produced a paper entirely using ChatGPT [34]. Prestigious scientific journals such as Nature and JAMA Network Science stated that they will not accept manuscripts generated by ChatGPT. In contrast, other journals ask authors to disclose the extent of ChatGPT’s use and to affirm their responsibility for the paper’s content [35,36,37]. Authorship guidelines distinguish between contributions made by humans and those made by ChatGPT. Specifically, authors are expected to provide substantial intellectual input. They must possess the ability to consent to co-authorship, thereby assuming responsibility for the paper or for the part to which they have contributed [38].

The ability of ChatGPT to compose scientific papers and abstracts in a manner indistinguishable from human output is remarkable. A study by Northwestern University (Chicago, IL, USA) highlighted this ability through a blind evaluation involving 50 original abstracts alongside their counterparts generated by ChatGPT from the original articles. These abstracts were randomly presented to a panel of medical researchers asking them to identify which were original and which were produced by ChatGPT. The outcome of this experiment was very intriguing since blinded human reviewers correctly identified only 68% of the ChatGPT-generated abstracts as such. Conversely, they mistakenly classified 14% of the actual human-written abstracts as being created by the chatbot. The challenge in distinguishing between authentic and AI-generated abstracts highlights ChatGPT’s skill in creating persuasive scientific narratives [39,40]. However, this raises critical concerns about the integrity of scientific communication. The fact that these AI-generated abstracts seem genuine and score high on originality according to plagiarism detection software introduces a paradox [20,41,42]. It suggests that while ChatGPT can produce work that is apparently innovative and unique, this may not necessarily reflect true originality or contribute to genuine scientific progress. This situation calls into question the reliability of using such software as the sole metric for originality and underscores the need for more nuanced approaches for evaluating the authenticity and value of scientific work. The reliance on AI for generating scientific content without critical oversight could risk diluting the scientific literature with works that, despite being original in form, lack the depth and rigor of human-generated research [43,44]

Notably, apart from the lack of responsibility for AI systems, some other ethical issues have been highlighted. In fact, it is well known that AI algorithms may be influenced by biases of healthcare data. Some other ethical implications in the use of AI systems concern the lack of transparency, data quality (i.e., the presence of “hallucinations”), and privacy vulnerabilities [45].

In particular, among the multitude of information generated by ChatGPT, a critical limitation arises from the inability to verify its data sources, as its responses are synthesized from an extensive dataset from the web without direct access to or citation of these sources showing a lack of transparency [18,19]. AI will never replace human revision. ChatGPT’s inability to distinguish between credible and less credible sources leads to transparency issues, treating all information equally. This differs from traditional research methods where source credibility can be verified. Users lack the means to judge information accuracy without external checks, highlighting concerns about the potential for misinformation and the necessity for external validation to ensure content reliability [24,46]

Furthermore, sometimes ChatGPT fabricates its references. Inaccurate references were reported as one of the three key features that guide the correct identification of human and AI-generated articles. In a single-blinded observer study, human and ChatGPT dermatology case reports were evaluated by 20 medical reviewers. One of the two selected case reports described a rare disease (posterior reversible encephalopathy syndrome) associated with pharmacological therapy. Human reviewers accurately identified AI-generated case reports in 35% of the reports. Three key features were reported by reviewers as essential for discrimination: poor description of cutaneous findings, imprecise report of pathophysiology, and inaccurate references [47]. One of the notable challenges with ChatGPT’s outputs is the phenomenon known as “artificial hallucination”, particularly evident in its provision of creative references. These hallucinations refer to information or data generated by the chatbot that do not accurately reflect reality, despite their realistic appearance. This issue is especially prevalent in references, where ChatGPT might cite sources, studies, or data that seem legitimate but do not exist or are inaccurately represented [48,49]. When questioned about liver involvement in late-onset Pompe disease, the chatbot provided details about the co-occurrence of the two conditions and suggested references to support its thesis. However, it is well known that the Pompe disease (Glycogen storage disease II, OMIM#232300) involves liver disease in infantile-onset forms, but hepatic features are unique or almost rare in the late-onset form [50]. Furthermore, the chatbot provided fabricated references to support its thesis [48,49]. These inaccuracies are not due to intentional misinformation but originate from the model’s design to generate responses based on patterns learned from its training dataset. In this context, it is essential to underline that ChatGPT does not have access to the PubMed database, so it cannot realistically search for references. Indeed, when a user requests ChatGPT to provide references supporting their responses, it fabricates credible but nonexistent references [51]. In a recent study, Gravel and coworkers evaluated 59 references provided by ChatGPT and retrieved that almost two-thirds of them were fabricated.

Interestingly, the fabricated references seemed real at first glance. About 95% (56/59) of reported references contained authors who published papers in the requested field, and 100% reported titles that seemed appropriate. Despite this truthfulness, 69% of references were fabricated [51]. Interestingly, change in the topic did not improve the truthfulness of references [52,53]. Another described ChatGPT hallucination is in the production of medical notes, sometimes fabricating patient features (i.e., it generates BMI score, without height and weight data) [23,54].

Notably, prompt engineering enhances the capabilities of pre-trained large language models and reports some methods to prevent LLM hallucinations [55]. In this scenario, some tips have been proposed to reduce the “temperature” of the interaction. In the LLM context, temperature is an indicator of the chatbot’s creativity in its answers [56]. When evaluating scientific fields, the temperature should be low. On the contrary, for creative tasks (i.e., poem generation) the temperature might be increased. Temperature might be changed in a raw API (application programming interface) interaction with LLM. However, ChatGPT can also be guided by providing instructions. For instance, the chatbot will provide more conservative answers when the question asks for a “direct” or “concise” response. Alternately, ChatGPT will provide a more creative or elaborate answer, when the user requests it to “be creative” or to “use imagination”. Similarly, changing the approach (i.e., from “one-shot learning” to “few-shot learning”) can improve chatbot performance [11]. Accurate guidelines to improve the input text and modify the GPT temperature are still lacking. ChatGPT proved to be useful in reviewing scientific manuscripts, being able to pinpoint their strengths and areas for improvement. Its skill set goes beyond content creation, including critical analysis and error detection, making it valuable for assessing medical data, even those it produces [21,22,23,24,57,58]. Its utility in these areas suggests that ChatGPT can assist researchers and medical professionals, offering a preliminary review that can optimize the revision process. However, it is imperative to integrate ChatGPT’s output with expert human revisions to achieve the highest scientific communication and medical accuracy standards. This integration becomes particularly crucial in rare diseases. ChatGPT may inadvertently introduce errors in medical documents related to these diseases due to the nuances and complexities involved. Therefore, any material prepared by ChatGPT, including drafts and preliminary analyses, should undergo a comprehensive review by specialists in the relevant field. This ensures that the final documents shared with patients or submitted to scientific journals are accurate, reliable, and reflect the current medical knowledge, avoiding potential misdiagnoses or misinformation.

## 3. Education

ChatGPT could improve the dissemination of knowledge by generating manuscripts in multiple languages. In some contexts, English could be an impediment, and ChatGPT can bridge the gap by generating copies of a manuscript in different languages. Similarly, in the conduction of cross-cultural research studies, it may support communication processes. Nevertheless, great attention should be paid to content, as chatbots can generate misleading or inaccurate content with the risk of causing misrepresentation instead of knowledge dissemination [59].

ChatGPT demonstrated a good impact in limiting misinformation derived from the internet on cancer myths. In fact, despite much harmful information available online about cancer [60], the chatbot demonstrated good accuracy in its response to cancer myths [61]. Similarly, in other contexts, ChatGPT was helpful in providing comprehensive information to patients, helping them to understand medical information and treatment options [62]. Undoubtedly, it is advisable to subject ChatGPT to specific training to maintain its ability and prevent the sharing of incorrect information. This is particularly true for cases in which it has been required to answer questions about rare diseases, for which the available information on the web may be limited. Fine-tuning of LLMs, such as ChatGPT, can improve their performance in specific fields [63]. The relevance of fine-tuning might promote its application in medicine, especially in rare disorder management.

The platform’s feasibility is one reason for its widespread diffusion. Other main strengths of ChatGPT are in the form and accessibility of the platform. The user–chatbot interaction is straightforward and mimics a dialogue. Not all information provided is accurate, and mistakes are difficult to detect because of the chatbot’s linguistic ability. Correct and incorrect sentences are reported entirely appropriate for the context. For these reasons, ChatGPT could be applied to provide and explain basic medical information and treatment options, even in rare disorders, but it should be used with caution in other cases.

In this context, it is essential to note that young people are prone to using online resources instead of seeking help through face-to-face methods [64]. Thus, ChatGPT is expected to become one of the most interrogated tools for every need. It has already become one of the most trusted online chatbots. Notably, the trust is greater for administrative tasks (i.e., scheduling appointments) and lower for management of complex medical situations (i.e., treatment advice) [65].

ChatGPT shows a certain ability to detect diagnosis and provide medical advice when evaluating medical scenarios. In detail, a study on 96 unique vignettes representing clinical cases with different features (scenarios, clinical histories, ages, races, genders, and insurance statuses) reported that ChatGPT offered safe medical advice, often without specificity [66]. The substantial safeness of the chatbot may support the care continuum but confirm its inability to replace medical judgment. As an example, a French study evaluated ChatGPT responses to a virtual patient affected by systemic lupus erythematosus (SLE) and asked for their treatment. The chatbot emphasized the need for medical evaluation but provided inconsistent information on hydroxychloroquine use during pregnancy and breastfeeding, as well as incorrect dosage suggestions. This highlights the risk of using ChatGPT’s responses without medical supervision [21]. Similar issues were noted with cardiovascular conditions, where it performed better on straightforward questions and case vignettes than on complex decision-making scenarios [67].

Likewise, ChatGPT performances on question resolution were confirmed to be high across different specialties. A recent study enrolled 33 physicians from 17 specialties to produce 180 medical questions. Each question was classified according to difficulty levels (easy, medium, and hard) and was fed to ChatGPT. the accuracy and completeness of ChatGPT answers were evaluated: the median accuracy score was 5/6 (mean 4.4, SD 1.7), and the median completeness score was 3/3 (mean 2.4, SD 0.7) [68]. Interestingly, ChatGPT performances on rare and familial disorders (such as prolactinoma and age-related macular degeneration) were in line with other diseases, with a slightly improved result for age-related macular degeneration, for which there are many data available on the web. There were no significant differences according to the difficulty level of questions, except for completeness scores that reached a median of 2.5/3 (mean 2.4, SD 0.7) for complex answers [68].

It is widely recognized that language models, including ChatGPT, exhibit a frequency bias, performing better on concepts extensively covered in their training data and poorly on less represented topics [25]. Consequently, ChatGPT’s reliability varies with the availability of information online; it is more accurate for diseases well-documented on the internet and less so for those with limited information. For instance, ChatGPT’s insights on osteoarthritis and ankylosing spondylitis are notably more accurate than its information on psoriatic arthritis [26].

## 4. Medical Practice

### 4.1. Support in Communications

Mental health care is one of the most promising topics in which AI-driven chatbots have been developed. Several chatbots have been designed for psychoeducation, emotional support, and cognitive behavioral therapy [69,70,71]. These tools have been developed mainly because of known barriers in assessing treatment for mental disorders. Beyond the long waiting times and geographical limitations similar to other medical specialties, psychiatric patients have to also face the stigma surrounding mental health [72]. One of the main acknowledged advantages of ChatGPT is the ability to generate sentences similar to a human-like conversation. In medical practice, there are some contexts in which the doctor–patient relationship may complicate the administration of evaluation questionnaires. In particular, in psychiatry, many interfering factors, such as the physician’s voice, mood, and environment, can interfere with the patient’s assessment. An impersonal interface such as ChatGPT can support the administration of questionnaires with human-like dialogues, but without the human interfering factors [73]. Likewise, there are other contexts where medical knowledge is deficient in detecting rare disorders and associated risks. For example, it is well known that patients affected by Charcot-Marie Tooth disease should avoid some drugs which may accelerate the disease’s progression. Unfortunately, many patients remain undiagnosed [74], lacking appropriate management of their condition. The diffusion of friendly instruments such as ChatGPT, after complete training for rare disorders, might improve the diagnostic ability of general doctors in detecting rare disorders or, at least, patients that should require a deeper evaluation.

It is expected that, with appropriate training, ChatGPT might be applied to clinical practice. It has been proposed to schedule appointments, collect anamnesis, and write medical records [43]. In particular, a study conducted by Cascella and coworkers revealed that ChatGPT can correctly write a medical note for patient admission in an intensive care unit (ICU) based on provided health information (treatments, laboratory results, blood gas, respiratory, and hemodynamic parameters). As expected, the main limitation was the causal relationship between pathological conditions. In this context, authors reported undeniable usefulness of ChatGPT in summarizing medical information using technical languages, but they underlined the need to pay great attention to content that required medical expertise, such as the identification of causal relationships among conditions [43]. In clinical practice, many significant medical records are very time-consuming. Quickly composing these documents may improve communication among healthcare centers. ChatGPT demonstrated a remarkable ability in composing patients’ discharge summaries and operative notes [75]. These documents require expert revision, but the diffusion of these AI models’ language in medical centers may improve the time to produce these medical records, providing a quick and high-quality transition among healthcare centers [76]. Furthermore, ChatGPT’s ability to admit and learn from mistakes is very promising [23]. For example, in the operative note for a patient with age-related macular degeneration, ChatGPT correctly adjusted anesthesia details associated with intraocular ranibizumab injection [76].

### 4.2. Support in Diagnosis and Differential Diagnosis

In medical practice, AI technologies are entirely used to support the definition of diagnosis, prognosis, and assessment of therapeutic targets (i.e., in oncology, it can provide treatment suggestions based on MRI radiomics and aging-related diseases) [77,78,79]. Unlike specific AI technologies developed and trained for medical purposes, ChatGPT has been developed without specific medical training. It makes responses to user questions based on internet datasets, without distinguishing between reputable and non-reputable sources. For these reasons, blindly relying on ChatGPT suggestions is dangerous.

Dynamism is one of the most essential features of ChatGPT and other AI language models. In fact, while diagnostic tools, such as Isabel [25,80,81], contain clinical data for a considerable number of diseases, they are static and cannot evaluate some individual data. Typically, the case presentation is based on a list of signs and symptoms, making the submission of clinical cases inappropriate. The conversation model included in ChatGPT supports the dynamic presentation of the clinical case, improving efficiency and relevance. In a recent study on the accuracy of Isabel in finding the correct diagnosis in ophthalmic patients, it did not perform as well as ChatGPT. In particular, Isabel provided the correct diagnosis within the first 10 results in 70% of patients, while ChatGPT reached 100% within the first ten differentials [25]. Notably, the latter chatbot confirmed its results in rare and familial disorders, giving the correct diagnosis in both Behcet’s Disease and AMD. However, Isabel misdiagnosed both cases, respectively, as relapsing polychondritis and uveitis.

Even in infectious diseases, ChatGPT has been evaluated to test its ability in providing diagnostic or therapeutic advice correctly in non-chronic clinical cases. It shows a good but not optimal performance, reaching an average score of 2.8, where the rating spanned from 1 (poor, incorrect advice) to 5 (excellent, fully corresponding with the advice of infectious disease and clinical microbiology specialists) [15].

Interestingly, when compared with specialist and non-specialist physicians, ChatGPT performances were surprising. In a neurology study, 200 synthetic clinical neurological cases were fed into ChatGPT, asking for the five most probable diagnoses. The results were compared with answers from 12 medical doctors (six “experts”, neurology specialists, and six general medical doctors). The first (most probable) diagnosis given by ChatGPT was correct in 68.5% of cases. This result is surprising because the medical doctor group achieved only 57.08% (±4.8%), while the expert group achieved 81.58% (±2.34%). As expected, some clinical cases were misdiagnosed. In particular, 10 cases were classified as “unsolved” because all experts failed to provide the correct diagnosis [82]. Notably, among these cases, there are some genetic and rare disorders. The accuracy of the chatbot in recognizing rare genetic disorders or correctly answering about them is limited. For example, when asked about the relationship between mutations in SCN9A and autosomal dominant epilepsy, ChatGPT incorrectly gave positive responses [83,84,85]. This evidence strongly suggests that ChatGPT may be misleading in evaluating rare disorders, which should also be assessed by a geneticist and/or other specific clinical tools. The ability of ChatGPT (GPT-3.5 and GPT-4) to detect the correct diagnosis was very weak for rare disorders, while it was acceptable for common diseases [86]. Compared with medical doctors, ChatGPT reached the correct diagnosis in the first three responses in over 90% of typical cases, which is quite similar to results from medical doctors (in over 90% of typical cases, they identified the correct diagnosis in the first two responses). For rare disorders, the performance of both ChatGPT and medical doctors decreased: GPT-3.5 reached 60% within the first ten responses, GPT-4 reached 90% within the first 8–10 responses, and medical doctors solved 30–40% of cases with their first suggested diagnosis; for two of them, the diagnostic accuracy increased to 50% within the first two suggested diagnoses [86].

The differences significantly increased when ChatGPT diagnostic performances were compared to a less experienced group, such as medical-journal readers. A study on clinical case challenges from New England Journal of Medicine revealed that GPT-4 provided the correct diagnosis for 22 of 38 clinical cases (57%), whereas the medical-journal readers chose the proper diagnosis among six provided options for 36% of cases [87].

**Figure 2:**
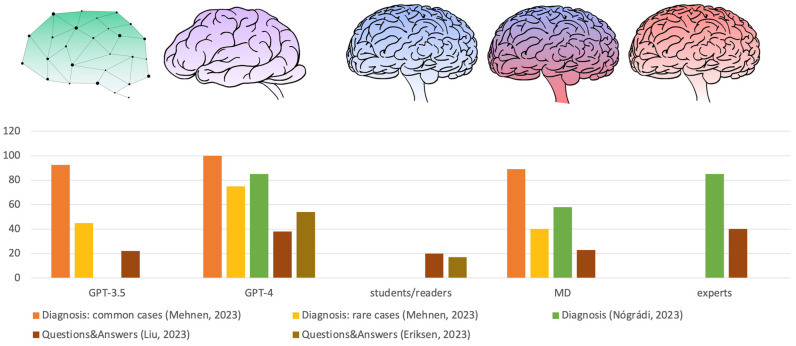
Comparative performance analysis. (Left to right: GPT-3.5, GPT-4, medical-journal readers/students, medical doctors (MDs), experts) [82,86,87,88].

ChatGPT is continuously evolving, and the evaluation of ChatGPT4 reliability shows a significant improvement if compared with ChatGPT 3.5. A recent analysis reported a relative safety of output information in more than 90% of responses (91% for GPT-3.5 and 93% for GPT-4), categorized out of a group of “so incorrect as to cause patient harm”. Unfortunately, the concordance between ChatGPT results and physician answers remains low (21% for GPT-3.5 and 41% for GPT-4) [46]. In a more specific context (neurosurgery), GPT-4 confirmed its better performance when compared with GPT-3.5. The percentage of consistent responses, according to guidelines and expert opinions, was 42% for GPT-3.5 and 72% for GPT-4.0 [79] (Figure 2).

### 4.3. Support in Treatment Advice

A study on ChatGPT’s reliability in reporting cancer therapies for solid tumors showed high scores. In particular, 51 clinical situations and 30 distinct subtypes of solid tumor were posed to ChatGPT, asking for therapies that can be used as first-line treatment. The chatbot results were compared with NCCN (National Comprehensive Cancer Network) guidelines. The accordance among responses was measured by searching ChatGPT-suggested therapies among first-level therapies listed in NCCN guidelines. In all circumstances, ChatGPT named therapies that may be used as first-line treatment for advanced or metastatic solid tumors. One point to consider is that some of the responses included alternate or preliminary drug names (i.e., Blu-667 for pralsetinib). Another important information is that the study evaluates general NCCN guidelines. However, recommendations may individually vary among patients [89,90,91]. In a consequential survey of the same field, but with different criteria for chatbot response evaluation, theauthors revealed that one-third of treatment recommendations also included one or more drugs non-concordant with NCCN guidelines. Moreover, ChatGPT recommendations changed depending on the question [92]. Similarly, in neuro-oncology, the ability of ChatGPT in adjuvant therapy decision-making was evaluated for glioma patients. Of them, 80% of patients were diagnosed with glioblastoma, a rare malignant brain tumor [93,94], and 20% had low-grade gliomas. Interestingly, when given the patient summary, the chatbot correctly recognized and classified the tumors as glioma, suggesting a tumor type (glioblastoma, grade II or III astrocytoma, etc.). While ChatGPT reported the need to modify treatment according to the patient’s preferences and functional status, no alternative therapies were listed, nor an alternative diagnosis [95]. Otherwise, the treatment suggestions were evaluated positively by a team of experts [95].

In summary, ChatGPT’s performance evaluation depended on the extensiveness of the knowledge and their availability on the web. In particular, the assessment of patient functional status was defined as moderate, maybe because of the small number of clinical trials available online [25]. In this scenario, it seems that the naïve ChatGPT can support multidisciplinary activities in neuro-oncology, but it requires complete training. For these reasons, Guo and coworkers created a trained version of ChatGPT, called neuroGPT-X, and evaluated it, comparing with naïve ChatGPT and leading neurosurgical experts worldwide. Despite its ability to support the neurosurgeons, the human expert’s evaluation remains necessary, mainly to ensure the safety and reliability of chatbot responses [96].

A recent study compared neurosurgery knowledge among chatbots (GPT-3.5 and 4.0) and neurosurgeons with different seniority levels. Fifty questions about neurosurgical treatments were submitted to the chatbot and neurosurgeons. The answers were evaluated by a team of senior neurosurgeons, who judged them as “consistent” or “inconsistent” with recommendations available in guidelines and evidence-based knowledge. The ability of GPT-3.5 was similar to that of neurosurgeons with low seniority, while GPT-4.0 ability was similar to that of neurosurgeons with high seniority [88].

Another study evaluated the reliability of ChatGPT in reporting potential dangers associated with drug–drug interactions [97]. Juhi and coworkers asked the chatbot, “can I take A and B together?”, where A and B represent two drug names, and, successively, “why should I not take A and B together?”. They tested 40 drug–drug interaction pairs, and only one interaction was unrecognized as dangerous. Interestingly, when the authors submitted the second question to the chatbot (“why should I not take A and B together?”), it corrected the first wrong answer. As expected, answers to the second question were less precise, describing molecular pathways but frequently with no conclusive facts supporting the drug–drug interaction [97]. In this context, ChatGPT confirmed its ability to recognize mistakes [23].

## 5. Discussion

In this review, medical applications of ChatGPT have been reviewed, from support in drafting medical records to more specialistic purposes, such as medical diagnosis, differential diagnosis, and treatment. In all its applications, the chatbot reported good results, still requiring close human supervision. Besides “artificial hallucinations” that may compromise the quality of medical records, many other inaccuracies have been noted. In particular, in the diagnostic path, it suffers from the absence of a dialogue, although the language simulates it. The chatbot bases its clinical assessment on information provided without the ability of exploring the clinical context posing further questions. In this scenario, GPT, as well as other LLMs (such as Gemini), is far from replacing human qualities in medical practice [98]. Nonetheless, GPT represents a technology with enormous potential that is rapidly growing. In fact, the new era of AI chatbots has the ability to analyze images. Apart from GPT-4, Gemini (Google’s AI) and LlaMa (Meta’s AI) are promising AI chatbots with image analysis capabilities. To date, this ability seems to be far from application in clinical practice [3], because the evaluation of medical images is complicated by many factors. Nevertheless, the integration of chatbots with existing AI systems, such Face2Gene, might improve the ability to recognize rare disorders [99]. Furthermore, although GPT demonstrated a good, even if non optimal, performance in recognizing rare disorders, the chatbot achieved better results than other diagnostic tools [25]. In this scenario, it is predictable to expect that a short training of GPT might improve its performance even in rare disorders. One of the main problems in diagnosing and treating patients with rare disorders is the lack of expert physicians. In particular, to recognize a patient potentially affected by a rare disorder, it is necessary that a medical doctor identifies clinical signs and symptoms and refers the patient to a specialistic healthcare unit. Unfortunately, the number of medical doctors able to correctly identify these signs and symptoms is limited. This is evident from the significant number of rare disorders that are still undiagnosed [74]. Adequate training in a user-friendly platform such as ChatGPT might improve the ability of medical doctors to recognize signs and symptoms associated with a rare disorder. Similarly, the chatbot may simplify the evaluation of familial history, making easier the detection of familial genetic disorders. ChatGPT interrogation could be a valid support to screen and identify families requiring a more precise genetic evaluation. To date, the only survey conducted on potential applications of ChatGPT among genetic counsellors reported a skeptical attitude. The most common concern about using ChatGPT was the risk of incorrect answers (82.2%) [100]. As seen in other medical contexts, ChatGPT is used in clinical practice by almost one of three interrogated genetic counsellors for drafting medical documentation (consult notes, result letters, and letters of medical necessity). A tiny percentage of genetic counsellors reported using the chatbot for clinical information on rare disorders (14.1%) or for differential diagnosis (8.6%).

As evident, the ChatGPT consultation cannot substitute for the medical doctor’s evaluation. One of the most critical shared limits of ChatGPT is the inability to correctly investigate the patient’s clinical features. It may seem obvious, but in clinical practice, the asking process is fundamental in the diagnostic path. ChatGPT has been tested with clinical cases ready for evaluation. Conversely, in the clinical practice, patients manifest one or more signs/symptoms of disease, and often, the physician has to ask for other sings/symptoms to complete the clinical picture of the disease [86,101]. The ability to ask the right question is one of the main ChatGPT limits, requiring medical intervention to translate the clinical phenotype to a case vignette ready for chatbot evaluation [101].

Nevertheless, it is essential to note that ChatGPT does not claim to be a doctor, nor to replace one. In many cases, the chatbot answers with a disclaimer about “I am not a doctor, but” [24]; in other cases, it advises the patient to consult professional healthcare for evaluation on the necessity of medication. For example, in an assessment of theoretical psychological cases with sleep disorders, ChatGPT suggested a treatment plan based on non-pharmacological interventions [102]. This answer is not only in line with current guidelines [103], but it is also safe for the patients, because the suggestion of a pharmacological treatment was strictly correlated to the necessity of a medical consultation [102].

The immediate application of AI represents a significant technological advancement in the statistical analysis of merged datasets. Our prior experience in biostatistics within the field of ophthalmic genomics indicates that similar outcomes could have been achieved more rapidly and with smaller cohorts of case–control samples [104,105,106]. This suggests that AI not only enhances efficiency but also reduces the need for extensive sample sizes.

In summary, the evaluation of ChatGPT in the clinical practice of rare disorders demonstrated a good potential. In detail, research and diagnostic applications of ChatGPT as a support for healthcare professionals retrieved excellent results, with similar accuracy for common and rare disorders. In this context, it is well known that rare disorders are less frequent than patients affected by common disorders. Notably, internet information did not follow this tendency. In particular, web spaces about rare disorders are very widespread. Online available sources create chatbot datasets, so rare disorders might be equally represented in these datasets when compared with common disorders. This is a hypothesis that might explain the high accuracy of ChatGPT even in uncommon diseases. Anyway, whatever the reasons at the bases of these GPT performances, it is expected that specific training on rare disorders, from clinical presentation to progression, available treatments, and risks for family members, will improve the ability of the chatbot to support not only medical doctors, but also experts. The widespread adoption and tailored training of ChatGPT could significantly support medical doctors’ ability to identify patients with rare disorders, potentially reducing the number of undiagnosed cases. ChatGPT may become a routine tool in clinical practice, not as a substitute for medical evaluation but as an aid in assessing patients. However, it is essential to emphasize that while ChatGPT can offer valuable insights, its recommendations and corrections must be evaluated in conjunction with expert human judgment.

In the evolving landscape of healthcare, there is a pressing need to integrate specialized training programs into the medical education curriculum, aimed at equipping the forthcoming generations of medical professionals with the proficiency required to utilize artificial intelligence (AI) programs effectively. These AI programs, when translated as routine clinical tools, have the potential to significantly improve the efficiency, accuracy, and cost-effectiveness of medical services.

Complete familiarity and competence in leveraging AI technologies can ensure that future doctors are well-trained to navigate the complexities of modern healthcare, optimizing patient care. This strategic introduction of AI into medical training not only aligns with the technological advancements of our time but also underscores a commitment to advancing the quality and sustainability of healthcare practices.

## Data Availability

No new data were created or analyzed in this study. Data sharing is not applicable to this article.
